# Building sustainable research capacity at higher learning institutions in Tanzania through mentoring of the Young Research Peers

**DOI:** 10.1186/s12909-021-02611-0

**Published:** 2021-03-17

**Authors:** Emmanuel Balandya, Bruno Sunguya, Daniel W. Gunda, Benson Kidenya, Tumaini Nyamhanga, Irene K. Minja, Michael Mahande, Blandina T. Mmbaga, Stephen E. Mshana, Kien Mteta, John Bartlett, Eligius Lyamuya

**Affiliations:** 1 Muhimbili University of Health and Allied Sciences, Dar-es-salaam, Tanzania; 2 Catholic University of Health and Allied Sciences, Mwanza, Tanzania; 3 Kilimanjaro Christian Medical University College, Kilimanjaro, Tanzania; 4 Duke University, Durham, North Carolina USA

**Keywords:** Young researcher, Vertical mentoring, Peer-to-peer mentoring, Community of young research peers

## Abstract

**Background:**

Sustainability of research culture in Sub-Saharan Africa is threatened in part by the lack of a critical mass of young researchers with the requisite skills and interest to undertake research careers. This paper describes an intensive mentorship programme combining hierarchical (vertical) and peer-to-peer (horizontal) mentoring strategies among young researchers in a resource limited setting in Sub-Saharan Africa.

**Methods:**

A consortium of three partnering large Tanzanian health training institutions (MUHAS, CUHAS and KCMUCo) and two collaborating US institutions (UCSF and Duke University) was formed as part of the five-year Transforming Health Professions Education in Tanzania (THET) project, funded by the NIH through Health Professional Education Partnership Initiative (HEPI). Within THET, the Community of Young Research Peers (CYRP) was formed, comprising of inter-professional and cross-institutional team of 12 Master-level Young Research Peers and 10 co-opted fellows from the former MEPI-Junior Faculty (MEPI-JF) project. The Young Peers received mentorship from senior researchers from the consortium through mentored research awards and research training, and in turn provided reciprocal peer-to-peer mentorship as well as mentorship to undergraduate students.

**Results:**

At the end of the first 2 years of the project, all 12 Young Peers were proceeding well with mentored research awards, and some were at more advanced stages. For example, three articles were already published in peer reviewed journals and two other manuscripts were in final stages of preparation. All 12 Young Peers participated in CYRP-wide thematic training workshops on mentoring and secondary data analysis; 11 had undertaken at least three research training short courses in identified areas of need; 9 joined at least one other ongoing research project; 5 made at least one scientific presentation, and 5 participated in at least one submitted grant application. Half of the Young Peers have enrolled in PhD programmes. A collective total of 41 undergraduate students were actively mentored by the Young Peers in research.

**Conclusion:**

The CYRP has demonstrated to be an effective model for dual vertical and horizontal mentorship in research to young investigators in resource-limited settings. This model is recommended to educators working on developing research competence of early career researchers, particularly in Sub-Saharan Africa.

## Background

Strengthening research capacity at universities in Sub-Saharan Africa is key to socio-economic development [1, 2]. In this endeavor, both faculty and students need to be empowered to be proficient across the whole spectrum of research undertaking, including conceptualization of priority research problems, development of fundable research proposals, ethical data collection, data analysis, manuscript writing, and dissemination [3]. Since formal teaching of research courses to undergraduate and postgraduate students is often inadequate [4], experiential learning and mentorship in the institutions of higher learning is increasingly seen as a high impact strategy for inculcating a research culture among young faculty and students [5]. According to Lev et al [6]: “*Mentoring occurs when a senior person or mentor provides information, advice, and emotional support to a junior person or student over a period of time*”. Although the nature of mentoring relationships depends on institutional context, the ultimate goal is to enable the faculty and students be competent in conducting research, dissemination of research findings, and knowledge translation [7, 8].

The literature identifies two main forms of mentorship, namely: hierarchical and peer mentoring [5]. Whereas hierarchical mentoring involves individuals from two different social positions, such as a faculty and student [9]; peer mentoring entails a situation whereby a more experienced faculty or student helps a less experienced colleague improve overall performance by supporting and advising the mentee [7]. In addition, in peer mentoring, mentors and mentees are roughly of equal age and power – which enhances reciprocal learning and psychosocial support [10, 11].

The Transforming Health Professions Education in Tanzania (THET) project, funded by the US National Institutes of Health (NIH) through Fogarty International Center in 2018–2023, is part of the Health Professional Education Partnership Initiative (HEPI). THET is a partnership of three health universities in Tanzania, namely the Muhimbili University of Health and Allied Sciences (MUHAS) as the prime; collaborating with Kilimanjaro Christian Medical University College (KCMUCo), and Catholic University of Health and Allied Sciences (CUHAS); and two partnering US institutions which are the University of California, San Francisco (UCSF) and Duke University; with the aim to use innovative educational strategies to transform health education geared to produce health professionals who are competent and ready for interprofessional practice whenever they are. Specifically, the three aims of THET are to (1) foster competency-based education, (2) build supportive academic environments for education and research, and (3) enhance communication with stakeholders. One of the major goals of the second aim of THET is to mentor and support junior faculty to successfully work together across their professions in conducting research and obtaining funding through creation of a mentored Community of Young Research Peers (CYRP).

The THET’s CYRP innovatively applies both hierarchical and peer mentoring to achieve the following objectives: groom young researchers for succession; promote peer-to-peer mentoring among young researchers; promote research training, mentorship, innovation and output at higher learning institutions; promote new approaches in research and re-defining priority research needs; and strengthen multidisciplinary research collaborations, especially in HIV/AIDS which was the main priority identified in the THET project.

The purpose of this paper, therefore, is twofold. One, to present the THET project’s research mentorship conceptualization, implementation, and outcomes during the first 2 years of the THET project using descriptive statistics. Two, to enlighten about opportunities gained and lessons learned through implementing this component of the project.

## Methods

### Constituting the CYRP

*Senior Leaders:* The two most senior researchers (a professor from MUHAS and a professor from Duke University) participated in the grant application process and had indicated their main interest in the second sub-aim of aim 2 of THET on research capacity building (Sub-Aim 2.2). For effective research mentorship of the CYRP, it was felt prudent to increase the number of senior researchers. From each of the three partnering Tanzanian institution, one Institutional Leader for Sub-Aim 2.2 and one Senior Faculty were identified based on the following criteria: academic positions of senior lecturer or above, expertise in HIV/AIDS research, basic sciences, implementation sciences or socio-behavioral sciences and willingness to mentor Junior Faculty researchers. Later on, two additional senior researchers were added based on their expertise in research methodology and data analysis. Collectively, the 10 senior faculty constituted the team of Senior Leaders for the CYRP.

*Young Research Peers:* Selection of the junior faculty for the young research peer positions was a competitive process. An application form was prepared by the lead institution and posted on the websites of each of the three partner institutions. Altogether, 20 applications were received, and these were scrutinized and subsequently twelve junior faculty, four from each institution, were selected after meeting the set criteria of aged 40 years or below, track record of conducting research, especially in HIV/AIDS and belonging to the Schools of Medicine and Nursing.

*MEPI-JF Fellows:* It was also proposed and agreed to co-opt ten fellows of the completed Medical Education Partnership Initiative-Junior Faculty (MEPI-JF) project, an approach that would provide an opportunity to show synergy between MEPI-JF and HEPI, both of which are funded by PEPFAR through the NIH’s Fogarty International Center (FIC).

### Structured roles and responsibilities of young peers

#### Own governance

This was achieved through physical meetings and scientific training through video teleconferences. The first physical meeting of the CYRP was held at MUHAS during the second half of project Year 1 in February 2019. During this meeting, members of the CYRP introduced to each other and the Senior Leaders guided them in selecting their Overall Peer Leader through a democratic process. The elected President of the CYRP became the spokesperson for the Community, coordinated the biweekly scientific teleconference sessions and effectively linked the Community with the THET Project leadership.

#### Research mentorship

Each Young Peer was assigned primary research mentor to guide him/her through proposal development, research implementation and dissemination of study findings. Biweekly videoconference sessions were scheduled. During the first year, video teleconference sessions centered on presentation and discussion of research proposals developed by each of the Young Research Peer. There were also selected research training sessions where the Senior Leaders presented on identified topics as part of mentorship. Active participation of the Young Peers and MEPI-JF fellows was strongly encouraged. Through this process, intense Young Peer to Young Peer, MEPI-JF fellows to Young Peers and Senior Leaders to Young Peers interactions occurred. Significant inputs were also obtained and were used to inform the proposal development process. During the second year, video teleconference sessions centered largely on presentation and discussion of the implementation of research projects. During these sessions, peer-to-peer discussions were encouraged, and comments made facilitated the successful conduct of the CYRP research projects. Peer-to-peer critiques of manuscripts was also strongly encouraged within the Community. The goal of promoting peer-to-peer mentoring was to foster mutual support.

#### Mentorship of undergraduate students

During implementation of the mentored research projects, the young peers were directed to engage undergraduate students in their research projects and serve as their primary mentors. It was rightly conceived that this Young Peer to undergraduate student mentorship process would strengthen the mentorship skills on the part of Young Peers and at the same time enable the undergraduate students learn the basic aspects of research. In all three partnering institutions, undergraduate students have a mandatory research component. Young Peers in their capacity as junior faculty were also assigned undergraduate students for research mentorship by their respective departments. Young Peers introduced their research projects to undergraduate students assigned to them and those interested were included in the project.

#### Undertaking research training

At the beginning of the project, Young Peers were instructed to self-identify areas of research training needs with emphasis on research ethics, research methods, biostatistics and scientific writing. The Young Peers were encouraged and supported to attend the identified short courses which are offered at THET institutions. Besides short courses, research training also took place in the form of thematic trainings which were conducted once per year (mentoring workshop conducted in Year 1 and secondary data analysis conducted in Year 2).

#### Undertaking mentored research projects

Each Young Peer was asked to write a research proposal for funding by THET. The funding level for each research project was agreed to be USD 10,000. All 12 Young Peers wrote proposals under the guidance of their mentors and submitted them to their respective institutional Review Boards (IRBs) for ethical clearance. After ethical clearance of their research proposals, the Young Peers were given USD 5000 (half of the total research grant) to start research implementation during the second year and the remaining USD 5000 to be disbursed for research finalization during the third year of the THET project. Expenditure of the research funds followed directives on allowable and unallowable costs as per instructions in the US Department of Health and Human Services SF424 (R&R). At the beginning of the project, a presentation on Financial Compliance was given to the Young Peers to orient them to these requirements. Costs supported covered reagents, field expenses, software and other allowable items. Undergraduate students were funded indirectly through access to data and research resources made available to the Young Peers. Mentored research awards will also be given to the second cohort of twelve Young Peers who will be recruited in year 4 of the THET Project. After completion of their projects, the Young Peers will be guided to prepare research proposals for additional funding through major funding sources such as NIH, CDC, PEPFAR and others extramural sources.

#### Anonymous feedback from the young peers

In order to obtain unbiased response from the Young Peers, an online feedback tool was designed using “*Google Forms*” where Young Peers gave feedback on the mentoring process anonymously and without any fear. Feedback was sought on areas such as quality of annual physical meetings, quality of videoconference sessions, quality of meetings with institutional Senior Leaders and quality of meetings with primary research mentors. Responses to questions were ranked 0–5 with 0 being worst and 5 best.

### Data collection and analysis

Source of data for this paper included quarterly and annual implementation reports as well as progress reports and anonymous feedback from the Young Research Peers. Data has been analyzed descriptively in which information on some parameters was summarized numerically in frequencies and percentages. Young Peers anonymous feedback responses were ranked 0 (worst) to 5 (best) and are presented as means with standard deviations. Data is presented in tables and histograms. All methods were carried out in accordance with the relevant guidelines and regulations.

## Results

### Diversity in composition of the first cohort of the Community of Young Research Peers

Initially, eight (8) Senior Leaders were selected as planned. Later on, two (2) additional Senior Leaders were included into the team because of their expertise in quantitative research methodology and data analysis. Overall, the Senior Leaders for the CYRP comprised of ten (10) members, nine from the 3 partnering Tanzanian institutions and one from the collaborating US institution. The Senior Leaders had substantial expertise and experience in basic sciences (3), clinical sciences (3), implementation sciences (2) and behavioral sciences research (2) in HIV/AIDS. Academic ranks of the Senior Leaders included Senior Lecturer (5), Associate Professor (1) and Professor (4). These had the responsibility of primary oversight for the twelve selected Young Peers, four from each Tanzanian partnering institution. All Young Peers had two University degrees (Bachelor and Master). As per plan, most Young Peers were of medical and nursing background. Other attributes were as summarized in Table 1. The Young Peers elected their own leaders through a democratic process and took a center stage in planning and implementing the various activities within CYRP, including choice of topics for mentored research projects and the conduct of biweekly scientific videoconferences.

**Table 1:** Composition of the Young Research Peers

S/N	Attribute		JUNIOR FACULTY IN CYRP		
			MUHAS (***n = 4***)	CUHAS (***n = 4***)	KCMUCo (***n = 4)***
**1**	**Age**	≤35 years *n, (%)*	3 (75)	3 (75)	1 (25)
		36–40 years *n, (%)*	1 (25)	1 (25)	3 (75)
**2**	**Gender**	Male *n, (%)*	2 (50)	2 (50)	3 (75)
		Female *n, (%)*	2 (50)	2 (50)	1 (25)
**3**	**Discipline**	Medicine *n, (%)*	2 (50)	3 (75)	3 (75)
		Nursing *n, (%)*	1 (25)	1 (25)	1 (25)
		Bioethics *n, (%)*	1 (25)	–	–
**4**	**Academic rank**	Assistant Lecturer *n, (%)*	4 (100)	2 (50)	1 (25)
		Lecturer *n, (%)*	–	2 (50)	3 (75)

Besides the twelve Young Peers, ten MEPI-JF fellows from CUHAS and KCMUCo were co-opted to join the CYRP. All the ten MEPI-JF fellows were medical doctors with diversified specializations in epidemiology, basic sciences (physiology, parasitology) and clinical medicine (paediatrics, psychiatry, obstetrics and gynaecology, surgery and internal medicine). The MEPI-JF fellows were responsible for providing horizontal mentorship to Young Peers on self-governance, expertise in qualitative and quantitative research as well as implementation of the mentored research projects.

### Research output and individual growth of the young peers in the first 2 years of the project (2018–2020)

All Young Research Peers had acquired ethical clearance for their research projects and were actively involved with data collection by the end of the first quarter of project year 2. The spectrum of research projects conducted included ethico-legal and societal issues (ELSI), behavioural sciences research, basic sciences research, clinical research and secondary data analysis with the primary focus on HIV/AIDS (Table 2). By end of the second year of the project, three primary articles emanating from Young Peers research projects had been published in peer-reviewed journals and other manuscripts were in various stages of preparation (Table 2). Overall, 29 manuscripts are expected from the Young Peers.

**Table 2:** Young Peers Research Areas and Publications

S/N	Research Area	Manuscripts				
		No.	Title	Status	Affiliations of authors	Number of co-authors
YOUNG PEERS FROM MUHAS						
1	Ethical role of community advisory boards in HIV clinical trials	1	How do community advisory boards fulfil their ethical role in HIV clinical trials? A protocol for a systematic review of qualitative evidence	Published (Pancras G et al., BMJ, 2020. doi: 10.1136/bmjopen-2019-035368)	MUHAS, KCMUCo	Senior leaders = 2 Junior faculty = 4 Students = 0 Other collaborators = 0
		2	Exploring the ethical role of community advisory boards in HIV clinical trials in Tanzania: revisiting the fundamental ethical principles	Data collection ongoing	In progress	In progress
2	Tuberculosis, HIV in adolescents	3	Medical management of acute loss of vision in tuberculous meningitis: A case report.	Published (Amour M et al.*,* J Clin Tuberc Other Mycobact Dis, 2020. doi: 10.1016/j.jctube.2020.100145)	MUHAS, CUHAS, New Young University Langone Health	Senior leaders = 0 Junior faculty = 1 Students = 0 Other collaborators = 2
		4	Adherence to antiretroviral therapy and associated factors among adolescents living with HIV in Dar-es-Salaam	Data analysis	In progress	In progress
		5	Loss to follow up among Adolescents living with HIV attending care and treatment clinics in Dar-es-Salaam	Data analysis	In progress	In progress
3	Phone contact versus house visits for index partner HIV testing	6	Effects of methods of reaching index partners for HIV testing: Observational study in two regions of Tanzania	Data collection ongoing	In progress	In progress
		7	Perception of healthcare givers on methods of reaching index partners for HIV testing in two regions of Tanzania	Data collection ongoing	In progress	In progress
4	Effects of Aspirin on HIV disease progression	8	Levels of biomarkers of immune and platelet activation in arv drug naive HIV infected individuals	Data collection ongoing	In progress	In progress
YOUNG PEERS FROM KCMUCo						
1	Spectrum of cancers in HIV infected patients	9	Cancer spectrum in HIV patients: A zonal hospital experience in Tanzania	Published (Mremi A et al*,* Cancer Treat Res Commun, 2020. doi: 10.1016/j.ctarc.2020.100213	KCMUCo, Duke University	Senior leaders = 2 Junior faculty = 1 Students = 3 Other collaborators = 1
		10	Capacity of Diagnostics and Treatment of Cancer in HIV-infected patients: A single hospital experience in Northern Tanzania	In preparation	In progress	In progress
		11	Prevalence of risk factors for cancer among HIV-infected patients in a referral hospital in Tanzania: a retrospective hospital-based study	Data analysis	In progress	In progress
		12	Incidence and profile of cancer among HIV-infected patients compared with the general population in a tertiary hospital in Tanzania, 2009–2019	Data analysis	In progress	In progress
		13	HIV-associated cancer in Tanzania: progress, challenges, opportunities	In preparation	In progress	In progress
		14	HIV-associated lymphomas: Clinico-pathological study from Tanzania	Data collection ongoing	In progress	In progress
		15	Cancer mortality and trends among HIV-infected inpatients in Tanzania	Data analysis	In progress	In progress
2	Effect of HIV and HAART on bone turnover	16	Systematic review of the effect of HIV-infection, combined antiretroviral therapy on bone turnover markers (BTMs) and bone mineral density in people living with HIV	Data collection ongoing	In progress	In progress
		17	Factors associated with increase in bone turnover markers among people living with HIV (PLWHIV) attending clinic at KCMC	Data collection ongoing	In progress	In progress
		18	Bone turnover markers and mineral density after 12 months on HAART.	Data collection ongoing	In progress	In progress
3	Asymptomatic bacteriuria in HIV infected individuals	19	Bacteriological profile and sensitivity pattern of isolates causing bacteriuria in people living with HIV	Data collection ongoing	In progress	In progress
4	Uptake of cervical cancer screening among women with HIV	20	Barriers and factors associated with low uptake of cervical cancer screening among women living with HIV at tertiary hospital, northern Tanzania	Data collection ongoing	In progress	In progress
		21	Role of awareness on cervical cancer screening uptake among women living with HIV at tertiary hospital, northern Tanzania	Data collection ongoing	In progress	In progress
YOUNG PEERS FROM CUHAS						
1	Dietary pattern and metabolic syndrome in patients with HIV	22	Metabolic syndrome in patients with HIV attending BMC care and treatment centre: Prevalence and associated factors	Data collection ongoing	In progress	In progress
		23	Dietary patterns and the factors associated with food choices in patients with HIV attending BMC care and treatment centre	Data collection ongoing	In progress	In progress
2	Pregnancy and post-partum mental disorders	24	Prevalence and correlates of depression among women attending ancs in mwanza Tanzania	Data analysis	In progress	In progress
		25	Perinatal anxiety among women attending antenatal clinics in mwanza tanzania	Data analysis	In progress	In progress
3	Drug adherence counselling in patients with HIV	26	Barriers to implementation of intensified adherence counselling among nurse counsellors at HIV Care and treatment clinic in Mwanza City	Data collection ongoing	In progress	In progress
		27	Proportion of people living with HIV who have achieved viral suppression and factors associated with the virological failure after 3 months of intensified adherence counselling among HIV patients with suspected virologic failure on first line ART attending HIV care and treatment clinic in Mwanza City	Data collection ongoing	In progress	In progress
4	Multi-drug resistant pathogens in HIV	28	Prevalence and incidence of Multi Drug Resistant pathogens colonizing-infecting children in Mwanza, Tanzania	Data collection ongoing	In progress	In progress
		29	Prevalence and incidence of Multi Drug Resistant pathogens colonizing-infecting HIV infected children in Mwanza, Tanzania	Data collection ongoing	In progress	In progress

**Fig. 1:**
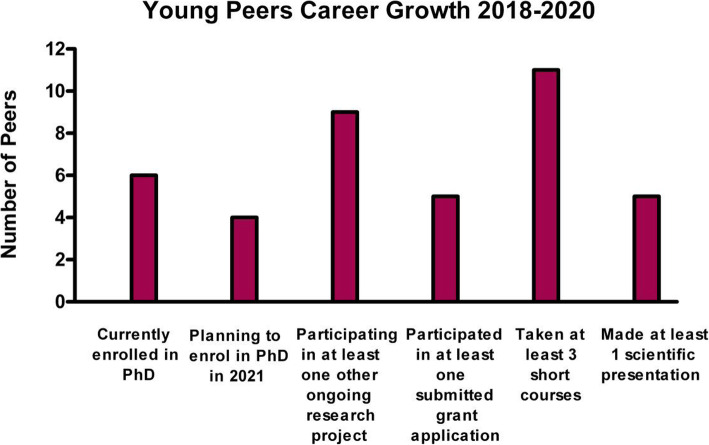
Young Peers career growth 2018–2020. A number of academic and scientific growth milestones were achieved by the Young Peers in the first two years of the project

Besides progress made in mentored research projects, the Young Peers registered substantial growth in other aspects including research training; participation in other ongoing research projects, including studies investigating febrile deaths in Tanzania, prevalence of transfusion-transmitted infections, environmental enteropathy in HIV-associated diabetes mellitus and patient satisfaction with hospitalization services; participation in submitted grant applications; and making scientific presentations. With regards to research training, two thematic training sessions on mentoring and secondary data analysis were conducted in project years 1 and 2, respectively, for all Young Peers. Other short courses undertaken by individual Young Peers depending on areas of identified need included foundation course in epidemiology and biostatistics, sampling techniques, qualitative and quantitative research methodology, qualitative and quantitative data analysis, logistic regression, survival analysis in clinical research, research ethics, literature search, manuscript writing, grant writing, impact evaluation, result-based monitoring and evaluation, demographic and health survey, systematic review and meta-analysis, good clinical practice, introduction to reviewing genomic research as well as introduction to evidence-informed decision making. Further to personal career growth, 6 Young Peers had registered in PhD programmes during the first two years of the project and 4 more are on course to register for PhD in project year 3. Summary of all career development milestones is presented in Fig. 1.

### Horizontal peer-to-peer mentoring in the CYRP

Besides the stated contributions of the CYRP to the institutions and scientific community through publications, scientific presentations and grant applications, the CYRP has significantly influenced career growth of the young researchers across the three partnering institutions. Firstly, the CYRP provided an opportunity for growth in research organizational skills, imparting research leadership skills to the Young Peers. Further, through horizontal peer-to-peer mentoring, the Young Peers and MEPI-JF fellows were able to support each other in various aspects including critiquing of research proposals, assistance with data collection and analysis through shared expertise in qualitative and quantitative research methodologies, undertaking joint research trainings, joint publications, sharing of funding opportunities and encouragement for career growth, such as enrolment in PhD. Peers were also able to share practical problem-solving skills such as devising strategies for circumventing the impact of Coronavirus Disease 2019 (COVID-19) outbreak on research and negotiating protected time for research at parent institutions.

### Vertical mentoring of undergraduate students in the CYRP

**Fig. 2:**
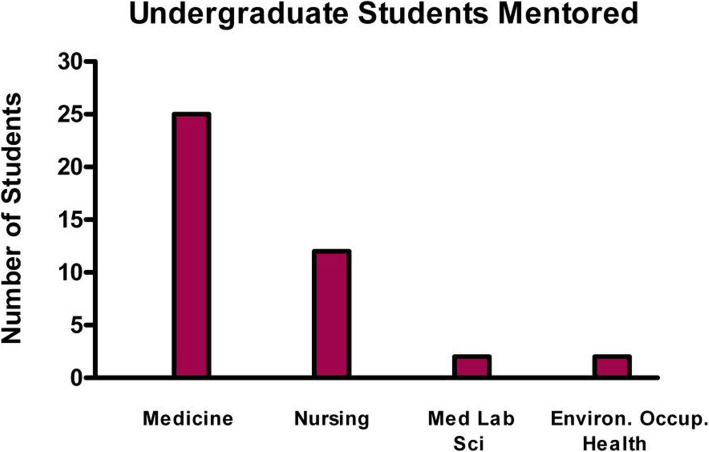
Undergraduate students mentored. 41 undergraduate students within and beyond Medicine and Nursing programmes were actively mentored by the Young Peers as part of the mentored research projects by the end of project year 2. Med Lab Sciences = Medical Laboratory Sciences; Environ. Occup. Health = Environmental and Occupational Health

In sustaining the vertical chain of research mentorship across the spectrum, the Young Peers were required to involve and mentor undergraduate students in their research projects. In total, 41 undergraduate students were incorporated in CYRP mentored research projects (Fig. 2). As per project goals, the undergraduate students were selected primarily from Doctor of Medicine and Bachelor of Science in Nursing degree programmes. In beginning to address the need for inclusivity across all programmes at institution, two undergraduate students came from Bachelor of Medical Laboratory Sciences and Bachelor of Environmental and Occupational Health. The scope of mentorship offered by Young Peers included guidance in developing research proposals for undergraduate elective research projects and nesting such research in CYRP mentored research projects. By the end of the second year of the project, three mentored undergraduate students appeared as co-authors in one of the publications.

### The effects and solutions to research interference due to COVID-19 outbreak

Following the surge of COVID-19 outbreak in Tanzania, recruitment of research participants for health research was temporarily put on hold by the Tanzania National Health Research Ethics Committee in April 2020. The ban was lifted off in May 2020 following encouraging trajectory of the epidemic in Tanzania, with directives to continue observing social distancing and use of personal protective equipment during research encounters. With the exception of a few research projects that were in advanced stages of data collection, most projects suffered during and after the lockdown period in a number of ways. Table 3 summarizes the ways in which projects by young research peers were affected and mitigation measures employed:

**Table 3:** Effects of COVID-19 and Mitigation Measures

S/N	Nature of Impediment	Number of Projects Affected	Mitigation Measures Employed
1.	Delays in data collection	8	1. Increase in recruitment sites 2. Extension of the duration for data collection
2.	Hiking up of prices and delays in delivery of procured research materials	3	1. Additional financial assistance from parent institutions 2. Archiving of samples for bulk testing
3.	Inability to hold physical meetings for research teams	8	1. Online meetings using *Zoom* and *GoToMeeting* platforms 2. Consultation via mobile phones

### Results of anonymous feedback from young peers on performance of the project

**Fig. 3:**
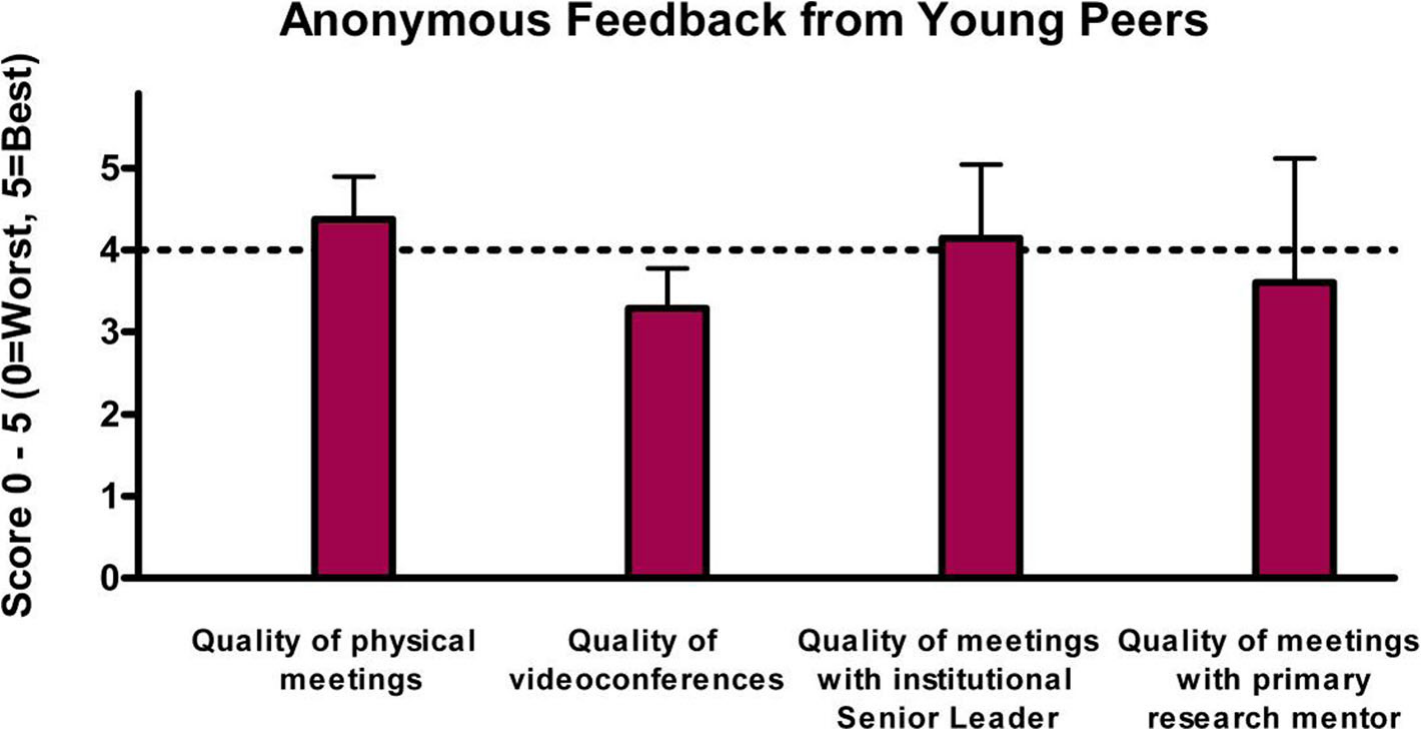
Results of anonymous feedback from Young Peers after first year of the project. Responses of Young Research Peers to questions evaluating their satisfaction with the conduct of the project. Bars indicate mean responses and error bars indicate standard deviation

Feedback from the Young Peers after first year of the project indicated that the Young Peers were satisfied with some areas in the conduct of the project, particularly on quality of the physical meetings and meetings with institutional Senior Leaders. However, the level of satisfaction with the quality of videoconference sessions and meetings with primary research mentors was lower (Fig. 3).

## Discussion

Nurturing the research careers of young investigators is paramount to sustainable growth and succession of research expertise in sub-Saharan Africa. The THET project sought to promote research at higher learning institutions by designing and implementing the Community of Young Research Peers (CYRP), a mentorship programme that was constituted by 5 pillars, namely: own governance, peer-to-peer mentoring, mentorship of undergraduate students, undertaking research training, and undertaking mentored research projects. This component of THET project has yielded several research outputs in the form of manuscripts and publications; and has provided an opportunity for growth in research organizational skills, impacting maturity in research leadership among the Young Peers.

The need for steering of the CYRP by a team of experienced investigators was realized at the inception of the project. We therefore constituted the CYRP with primary oversight by Senior Leaders from partnering institutions with experience in mentoring junior scientists. In line with the goal of THET to further HIV/AIDS research in Tanzania, the Senior Leaders had expertise in HIV/AIDS research, particularly in research methodology, basic sciences, implementation sciences and socio-behavioral sciences. The young faculty were similarly of diverse institutional and professional backgrounds, primarily medicine and nursing. Besides faculty, undergraduate students from various degree programmes were selected as lowest tier mentees in the CYRP. Collectively, the diversity of members in the CYRP provided a rich environment for sustainable growth of future investigators at different stages in research careers.

Although models of grooming the young investigators through vertical and horizontal mentoring strategies have been described previously [12, 13], our model is unique in that it combined both approaches. On one hand, the extended vertical mentorship arm ensured that the mentored young faculty get the opportunity to exercise mentoring of the undergraduate students in real time, and for the latter to start developing interest in research career early on. This created a mentored hierarchical pyramid which is essential to succession of research expertise. Besides vertical mentorship, the young researchers were also able to offer peer-to-peer mentoring among Young Peers themselves and between the Young Peers and MEPI-JF fellows. Of particular note, the MEPI-JF fellows were very resourceful to the Young Peers in offering expertise and experiences in study design, data collection and data analysis. Besides research, the Young Peers were also able to support each other in other areas such as ways of negotiating protected time, encouragement for enrolling in PhD programmes and the sharing of ideas on combating COVID-19 challenges. The Young Peers exercised own governance which further fostered their growth in research leaderships and organization. This model prepared young faculty early in on in undertaking their mentoring role and cultivating healthy relationships with colleagues along and across academic ranks, elements that are critical for successful mentorship [14–16]. Overall, our model of mentorship was unique in its design and implementation which enhances support between senior and junior faculty, between peer junior faculty, between junior faculty and students, and between student mentees.

As a testament to the effectiveness of our model of mentorship, the Young Peers had achieved substantial milestones in the first 2 years of the project. Notably, three research articles have been published and two other manuscripts are in final stages of preparation. The Young Peers shared authorship in one of the published articles. Most Young Peers had taken at least three research training short courses and six have newly enrolled in PhD programmes. Young Peers were beginning to broaden their research careers by involvement in other ongoing research projects and grant applications. This trajectory of career growth is remarkable and holds promise to great successes in the future.

The response of the group and parent institutions to COVID-19 was remarkable. In addressing the reduction in participant accrual rates and the need for social distancing, the Young peers responded by a myriad of measures ranging from protocol amendments to include additional data collection sites and using online platforms in conducting team meetings. Institutions were forthcoming and assisted in toping-up research funding to cover for the hiking prices of research materials. In direct response to COVID-19, one of the Young Peers partnered with colleagues on a side project to investigate the in-vitro filtration efficiency of cloth face masks, article currently under peer review [17].

Grooming of the young researchers through research groups is not without risk of academic inbreeding [18, 19]. In our group, the untoward effects of academic inbreeding were mitigated by fostering interaction of the young peers with the global scientific community through inclusion of an international senior leader, invited presentations by international collaborators, scientific collaborations with international research partners and participation in international scientific conferences. One peer published an article with an international collaborator outside of the consortium.

Despite milestones achieved, there were a number of challenges that occurred during the first 2 years of implementation that needed improvement. Firstly, there were delays in disbursement of funds for project activities. The financial officers at partner institutions have been working on streamlining the invoicing systems in order to improve on the speed of transfer of funds. Secondly, the amount of research funding disbursed to Young Peers is limited. In addressing this, the Senior Leaders have been active in soliciting and sharing with Young Peers various fellowship opportunities that may provide supplemental funding for research. Thirdly, the lack of protected time for research has been a major bottleneck for both Young Peers and Senior Leaders at partnering institutions due to competing roles in teaching and administration. This has been partly mitigated by encouraging Young Peers to enroll into PhD programmes where there is more time protection. Currently, six Young Peers are enrolled in PhD and the remaining ones have been encouraged to enroll in a near future. The fourth challenge, also raised by the Young Peers during anonymous feedback, was that the quality of videoconference sessions was suboptimal during the first year of the project. This was because of poor internet connectivity and the use of fixed videoconference facilities at the partnering institutions which had the disadvantage of requiring physical presence of the Young Peers and Senior Leaders at the institutions at the time of the sessions. This has been largely addressed by shifting meetings to the *Zoom* online platform which has the flexibility of connectivity using mobile devices and this has significantly improved on the quality and attendance at the sessions. The challenge of poor quality of meetings between Young Peers and their primary research mentors has been addressed by reminding research mentors to conduct frequent regular meetings with their mentees. Further, the project has also identified presentations at scientific meetings as an area requiring extra attention. In addressing this need, a training session on oral and poster presentations will be delivered to the Young Peers at the beginning of the third year of the project. Subsequently, Young Peers will be guided to submit abstracts for presentation at scientific conferences. In line with global adaptation to the COVID-19 pandemic, virtual conferences will also be targeted.

In the long run, the CYRP aims to further expand through selection of a second cohort of twelve Young Peers who will join the group in the fourth year of the THET project. The goal in the second cohort is to further diversify professions of the peers beyond medicine and nursing to include pharmacy, dentistry and public health. The spirit of inter-disciplinary and cross-institutional collaboration will be further emphasized by encouraging joint grant writing, joint research activities and joint authorship in scientific publications. The vertical and horizontal mentorship across hierarchies will continue to be emphasized, including implementing the research mentorship curriculum already in place. This way, the CYRP will foster sustainable research excellency at the partner institutions.

## Conclusion

Mentoring the early career investigators through inter-professional and cross-disciplinary Communities of Young Research Peers is a viable model for growth and succession of research expertise. This model is highly recommended to be adopted by educators striving to develop research careers of young investigators, especially in Sub-Saharan Africa.

## Data Availability

Data used in current study are available from the corresponding author on reasonable request.

## Abbreviations

AIDS: Acquired immunodeficiency syndrome
CDC: Center for diseases control
COVID-19: Coronavirus diseases 2019
CUHAS: Catholic university of health and allied sciences
CYRP: Community of young research peers
ELSI: Ethical-legal and societal issues
FIC: Fogarty international center
HEPI: Health professional education partnership initiative
HIV: Human immunodeficiency virus
IRB: Institutional review board
KCMUCo: Kilimanjaro christian medical university college
MEPI-JF: Medical education partnership initiative-junior faculty
MUHAS: Muhimbili university of health and allied sciences
NIH: National institutes of health
PEPFAR: President’s emergency plan for AIDS relief
THET: Transforming health professions education in tanzania
UCSF: University of California at San Francisco

## References

[1] Chu KM, Jayaraman S, Kyamanywa P, Ntakiyiruta G (2014). Building research capacity in Africa: equity and Global Health collaborations. PLoS Med.

[2] Mkony CA, Kaaya EE, Goodell AJ, Macfarlane SB (2016). Where teachers are few: documenting available faculty in five Tanzanian medical schools. Glob Health Action.

[3] Sawyerr A (2004). African universities and the challenge of research capacity Development1. J High Educ Africa.

[4] Munabi IG, Buwembo W, Joseph R, Peter K, Bajunirwe F, Mwaka ES (2016). Students’ perspectives of undergraduate research methods education at three public medical schools in Uganda. Pan Afr Med J.

[5] Collier P. Why peer mentoring is an effective approach for promoting college student success. Metrop Univ. 2017;28(3). 10.18060/21539.

[6] Lev EL, Kolassa J, Bakken LL (2010). Faculty mentors’ and students’ perceptions of students’ research self-efficacy. Nurse Educ Today.

[7] Colvin JW, Ashman M (2010). Roles, risks, and benefits of peer mentoring relationships in higher education. Mentor Tutoring Partnersh Learn.

[8] Crisp G, Cruz I (2009). Mentoring college students: a critical review of the literature between 1990 and 2007. Res High Educ.

[9] Kram KE, Isabella LA (1985). Mentoring alternatives: the role of peer relationships in career development. Acad Manag J.

[10] Angelique H, Kyle K, Taylor E (2002). Mentors and muses: new strategies for academic success. Innov High Educ.

[11] Terrion JL, Leonard D (2007). A taxonomy of the characteristics of student peer mentors in higher education: findings from a literature review. Mentor Tutoring Partnersh Learn.

[12] Dagher MM, Atieh JA, Soubra MK, Khoury SJ, Tamim H, Kaafarani BR (2016). Medical research volunteer program (MRVP): innovative program promoting undergraduate research in the medical field. BMC Med Educ.

[13] Bryant AL, Aizer Brody A, Perez A, Shillam C, Edelman LS, Bond SM, Foster V, Siegel EO (2015). Development and implementation of a peer mentoring program for early career gerontological faculty. J Nurs Scholarsh.

[14] Lewinski AA, Mann T, Flores D, Vance A, Bettger JP, Hirschey R (2017). Partnership for development: a peer mentorship model for PhD students. J Prof Nurs.

[15] Lindén J, Ohlin M, Brodin EM (2013). Mentorship, supervision and learning experience in PhD education. Stud High Educ.

[16] Abbott-Anderson K, Gilmore-Bykovskyi A, Lyles AA (2016). The value of preparing PhD students as research mentors: application of Kram’s temporal mentoring model. J Prof Nurs.

[17] Amour M, Mwanga H, Bwire GM. In-vitro filtration efficiency for selected face masks to bacteria with a size smaller than SARS-CoV-2 respiratory droplet. 2020. 10.21203/rs.3.rs-28705/v1.

[18] Horta H (2013). Deepening our understanding of academic inbreeding effects on research information exchange and scientific output: new insights for academic based research. High Educ.

[19] Horta H, Martins R (2014). The start-up, evolution and impact of a research group in a university developing its knowledge base. Tert Educ Manag.

